# Truncated forms of viral VP2 proteins fused to EGFP assemble into fluorescent parvovirus-like particles

**DOI:** 10.1186/1477-3155-4-13

**Published:** 2006-12-08

**Authors:** Leona Gilbert, Jouni Toivola, Outi Välilehto, Taija Saloniemi, Claire Cunningham, Daniel White, Anna R Mäkelä, Eila Korhonen, Matti Vuento, Christian Oker-Blom

**Affiliations:** 1 Department of Biological and Environmental Science and Nanoscience Center, P.O. Box 35, 40014 University of Jyväskylä, Finland

## Abstract

Fluorescence correlation spectroscopy (FCS) monitors random movements of fluorescent molecules in solution, giving information about the number and the size of for example nano-particles. The canine parvovirus VP2 structural protein as well as N-terminal deletion mutants of VP2 (-14, -23, and -40 amino acids) were fused to the C-terminus of the enhanced green fluorescent protein (EGFP). The proteins were produced in insect cells, purified, and analyzed by western blotting, confocal and electron microscopy as well as FCS. The non-truncated form, EGFP-VP2, diffused with a hydrodynamic radius of 17 nm, whereas the fluorescent mutants truncated by 14, 23 and 40 amino acids showed hydrodynamic radii of 7, 20 and 14 nm, respectively. These results show that the non-truncated EGFP-VP2 fusion protein and the EGFP-VP2 constructs truncated by 23 and by as much as 40 amino acids were able to form virus-like particles (VLPs). The fluorescent VLP, harbouring VP2 truncated by 23 amino acids, showed a somewhat larger hydrodynamic radius compared to the non-truncated EGFP-VP2. In contrast, the construct containing EGFP-VP2 truncated by 14 amino acids was not able to assemble into VLP-resembling structures. Formation of capsid structures was confirmed by confocal and electron microscopy. The number of fluorescent fusion protein molecules present within the different VLPs was determined by FCS. In conclusion, FCS provides a novel strategy to analyze virus assembly and gives valuable structural information for strategic development of parvovirus-like particles.

## Background

Canine parvovirus (CPV) is an autonomous, non-enveloped single stranded DNA virus with a diameter of 26 nm. The icosahedral T = 1 virion contains 60 protein subunits composed of three different polypeptide chains designated VP1, VP2, and VP3 [1-7]. VP1 is identical to VP2, but has 154 additional N-terminal amino acid residues. The VP3 protein is proteolytically cleaved from VP2 by removal of about 12 to 15 amino acids from the N-terminus [1,8]. The VP2 protein constitutes most of the capsid surface while VP1 represents only a small portion of the capsid composition. It has been shown that VP2 can assemble into capsid-like structures [9] and that the structure of empty CPV capsids had the first 37 residues not resolved structurally [9]. These structural proteins share a conserved β-barrel core domain that contains an eight-stranded, anti-parallel β-barrel motif consisting of two β-sheets in standard BIDG and CHEF arrangements common to many viral capsid proteins [10]. This domain accounts for one third of the amino acid content of each polypeptide. The other two thirds of the polypeptide sequence consist of four large loop insertions that form the surface of the virion.

Viral structures have been mainly characterized by X-ray crystallography and electron microscopy. Single molecule detection techniques have arisen for characterization of macromolecules moving persistently in non-denaturing physiological conditions. One such emerging method is fluorescence correlation spectroscopy (FCS) [11-14]. FCS characterizes interactions and molecular structures through the dynamic processes of molecules in solution. Statistical information is extracted from the averaged fluorescence intensity fluctuations of fluorescent molecules diffusing through a small measuring volume of less than one femtoliter [15,16].

In the present study, 14, 23 and 40 N-terminal amino acid deletions of the VP2 protein were fused to the C-terminus of EGFP. The corresponding proteins were produced in baculovirus infected *Spodoptera frugiperda *(S*f*9) insect cells, purified and then analyzed by FCS. Results indicated that the non-fused constructs deleted by 14, 23 and 40 amino acids, and fusion proteins of EGFP-VP2-23 and EGFP-VP2-40, as well as the non-truncated form of VP2 (EGFP-VP2), were able to form virus-like particles (VLPs) despite the presence of the bulky EGFP domain. Interestingly, the fluorescent mutant (EGFP-VP2-14) deleted by only 14 amino acids was not able to form similar structures.

## Results

### Expression of the CPV VP2 constructs in insect cells

**Figure 1:**
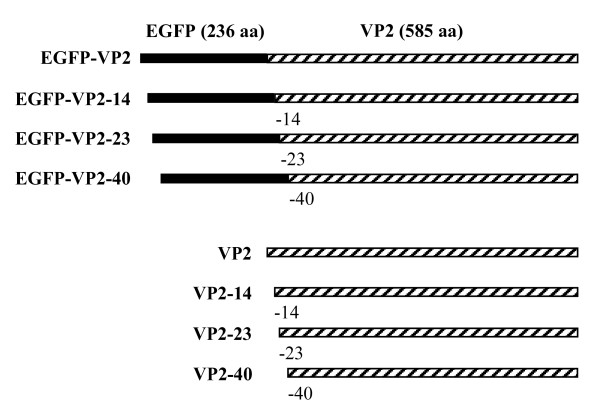
**Schematic representation of truncated forms of the canine parvovirus (CPV) structural protein VP2 and their fusions with the enhanced green fluorescent protein, EGFP**. The N-terminus of VP2 was deleted by 14, 23, and 40 amino acids and fused to the C-terminus of EGFP resulting in the constructs EGFP-VP2, EGFP-VP2-14, EGFP-VP2-23 and EGFP-VP2-40. The non-fused constructs VP2, VP2-14, VP2-23, VP2-40 are presented in the lower panel.

**Figure 2:**
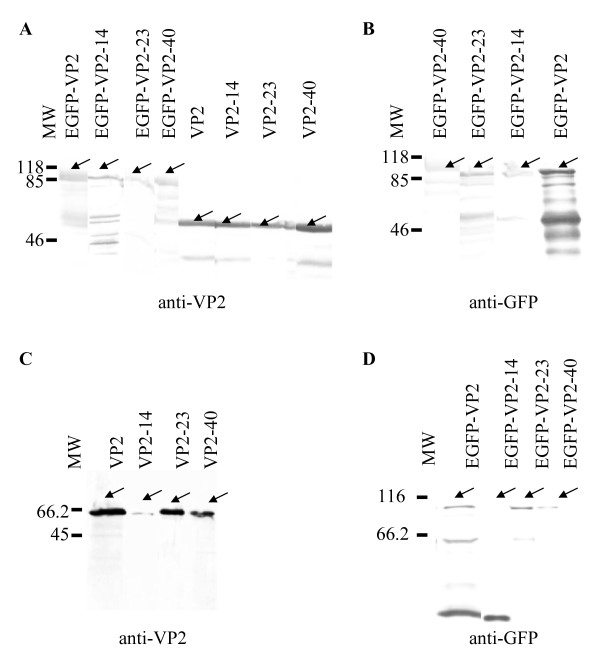
**Immunoblot analyses of the recombinant proteins**. Baculovirus infected *Sf*9 cells (A and B) expressing the fusion proteins EGFP-VP2, EGFP-VP2-14, EGFP-VP2-23 and EGFP-VP2-40, as well as the non-fused proteins VP2, VP2-14, VP2-23, and VP2-40. Sucrose gradient purified proteins are shown in C and D. Proteins were detected with anti-VP2 (A and C) and anti-GFP (B and D) antibodies. Arrows indicate the recombinant proteins of interest and the molecular weight markers (MW) are in kilodaltons (kDa; shown on the left).

CPV VP2 and the N-terminal deletions thereof VP2-14, VP2-23, and VP2-40 as well as the corresponding EGFP fusions EGFP-VP2, EGFP-VP2-14, EGFP-VP2-23, and EGFP-VP2-40 (Fig. 1) were produced in *Sf*9 cells infected with the respective recombinant baculoviruses *Ac*VP2, *Ac*VP2-14, *Ac*VP2-23, *Ac*VP2-40, *Ac*EGFP-VP2, *Ac*EGFP-VP2-14, *Ac*EGFP-VP2-23, and *Ac*EGFP-VP2-40. Expression of all recombinant proteins from cell lysates was confirmed by immunoblotting using anti-VP2 and anti-GFP antibodies and proteins of expected sizes (arrows) were identified (Figs. 2A and 2B). Particularly in the case of the EGFP-fusion constructs, some break down products could also be identified with both antibodies (Figs. 2A and 2B). For purification of the recombinant proteins, the infected cell lysates were exposed to sucrose gradient centrifugation and fractions of the recombinant proteins corresponding to assembled VLPs or capsid-like structures [17] were further analyzed by immunoblotting using anti-VP2 and anti-GFP antibodies (Figs. 2C and 2D). All proteins except the EGFP-VP2-14 fusion construct appeared to assemble into VLPs or resembling structures (Figs. 2C and 2D).

### Virus-like particle formation by truncated forms of CPV VP2

**Figure 3:**
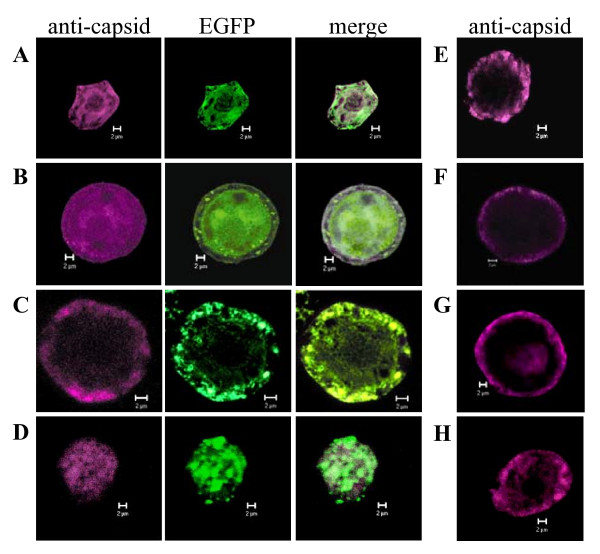
**Confocal imaging of *Sf*9 cells infected with recombinant baculoviruses**. (A) *Ac*EGFP-VP2, (B) *Ac*EGFP-VP2-14, (C) *Ac*EGFP-VP2-23, (D) *Ac*EGFP-VP2-40, (E) *Ac*VP2, (F) *Ac*VP2-14, (G) *Ac*VP2-23, and (H) *Ac*VP2-40. A monoclonal anti-capsid antibody (A4E3) for detection of assembled VP2 was visualized with AlexaFluor^® ^633-conjugated anti-mouse secondary antibody (violet), whereas EGFP (green) was imaged directly. Co-localization of the fusion partners in the merged images is shown in white. All images are single confocal midsections from single cells of approximately 0.7 μm in thickness. Bars 2 μm.

**Figure 4:**
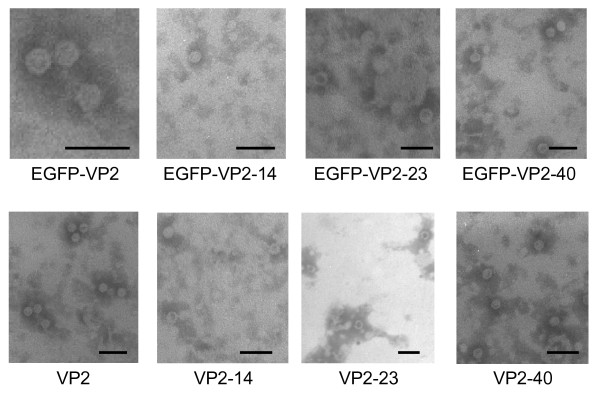
**Electron micrographs of negatively stained preparations**. Purified preparations from recombinant baculovirus infected *Sf*9 cells expressing EGFP-VP2, EGFP-VP2-14, EGFP-VP2-23, and EGFP-VP2-40 (upper panel) as well as VP2, VP2-14, VP2-23, and VP2-40 (lower panel) are shown. Bars 50 nm.

VLP formation was analyzed by confocal and electron microscopy studies. Confocal microscopy indicated that VP2 colocalized with their EGFP fusion partner, but that the colocalization was not complete (Fig. 3). The antibody used here was a mouse monoclonal anti-capsid antibody specific for an epitope present only on capsids and capsid-like structures (A4E3; kind gift from Dr. Colin Parrish, Cornell University, Ithaca, NY). Thus, the imperfect co-localization seen by confocal microscopy (Fig. 3) and the break down products in the western blots (Fig. 2) are most likely due to proteolysis of the EGFP-VP2 protein constructs as seen also in previous studies [18,19]. From the negatively stained EM micrographs of the VP2 proteins it was obvious, that VP2, VP2-14, VP2-23, VP2-40 as well as the fusion constructs EGFP-VP2, EGFP-VP2-23 and EGFP-VP2-40 were able to form structures resembling VLPs (Fig. 4). All particles appeared to have a diameter of approximately 26 nm, displaying a typical VP2 or wild-type CPV structure [18,19]. However, the EGFP-VP2-14 construct appeared not to assemble into similar structures (Fig. 4).

### Characterization of the fluorescent recombinant proteins

**Figure 5:**
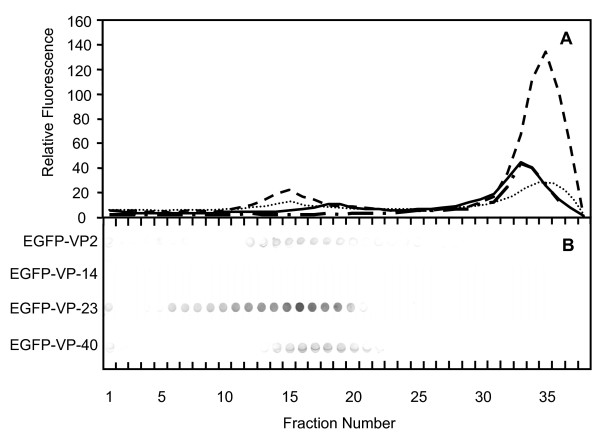
**Fluorescence measurements and dot blot analysis of insect cell lysates exposed to sucrose gradient centrifugation**. The samples were produced from *Sf*9 cells containing EGFP-VP2 (...), EGFP-VP2-14 (-.-), EGFP-VP2-23 (- -) and EGFP-VP2-40 (—) and the relative fluorescence determined (A). Dot blot analysis of the corresponding fractions using anti-capsid (A4E3) antibody detected by AP-conjugated goat anti-rabbit IgG (B). Fractions 1 to 37 (bottom to top) of the gradients are indicated below.

**Figure 6:**
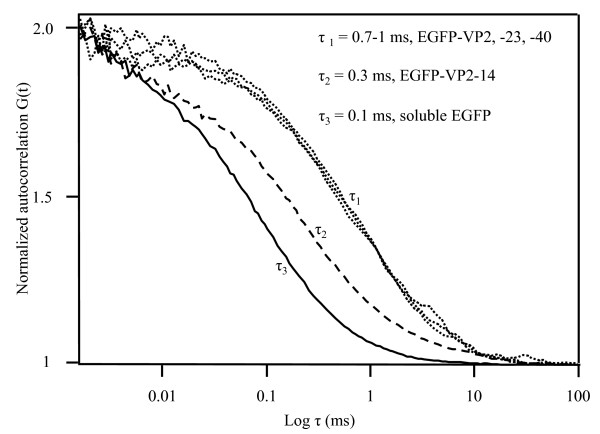
**Fluorescence autocorrelation curves of the fluorescent fusion protein constructs and EGFP**. Normalized autocorrelation curves of EGFP-VP2, EGFP-VP2-23, EGFP-VP2-40 (τ1), EGFP-VP2-14 (τ2) and soluble EGFP (τ3).

The recombinant proteins (EGFP-VP2, EGFP-VP2-14, EGFP-VP2-23, EGFP-VP2-40) were purified by sucrose gradient centrifugation prior to further examination. A total of 37 fractions were collected for each gradient and the relative fluorescence of each fraction was plotted against its fraction number (Fig. 5A). The fluorescence signal seen in this figure indicates that fractions near the top of the gradient (33–37) contain a large amount of non-assembled fusion proteins and/or free EGFP, whereas fractions 13–20 showed fluorescent bands visible to the naked eye for EGFP-VP2, EGFP-VP2-23 and EGFP-VP2-40 and were seen as peaks. A corresponding fluorescent band and peak for EGFP-VP2-14 was not seen, suggesting that this construct did not form fluorescent VLPs (fVLPs; Fig. 5A). Dot-blot analysis with monoclonal anti-capsid antibody (Fig. 5B) also showed peak patterns in fractions 13–20 for EGFP-VP2, EGFP-VP2-23 and EGFP-VP2-40. Again, no signal was seen for EGFP-VP2-14, indicating the lack of VLP structures for this construct. This characteristic distribution of recombinant viral proteins separated in a sucrose gradient, then observed by relative fluorescence and dot blot analysis (Fig. 5B) has been previously reported [20-23]. FCS autocorrelation analysis was performed using a one-component model. The diffusion time, being the time taken for a particle to travel through the 0.2 fl laser volume, was close to 1 ms for the EGFP-VP2, EGFP-VP2-23 and EGFP-VP2-40 fusions giving hydrodynamic radii of 14–20 nm (Fig. 6 and Table 1). For the EGFP-VP2-14 construct, the diffusion time was approximately 0.3 ms corresponding to a hydrodynamic radius of 7 nm (Fig. 6). EGFP diffused with a calculated hydrodynamic radius of 2 nm (Table 1). For comparison of the diffusion times in all constructs, normalized autocorrelation curves are shown in figure 6. Samples of all constructs, apart from EGFP-VP2-14, contained VLPs with a similar size to native CPV. Samples of EGFP-VP2-14 contained smaller particles, too small to be VLPs.

**Table 1:** Mobility of fluorescent canine parvovirus recombinant proteins.

Particle	τ_D1 _(ms)	τ_D2 _(ms)	*D*1 (m^2^s^-1^)	No urea, Rh_D1 _(nm)	6 M urea, Rh_D2 _(nm)	Nf
EGFP-VP2	0.7 ± 0.3	0.3 ± 0.1	1.4 × 10^-11^	17.0 ± 7.0	8.5 ± 2.7	9.5 ± 0.7
EGFP-VP2-14	0.3 ± 0.0	0.2 ± 0.1	3.6 × 10^-11^	6.7 ± 0.9	3.9 ± 2.3	2.0 ± 0.1
EGFP-VP2-23	0.8 ± 0.2	0.4 ± 0.1	1.2 × 10^-11^	20.0 ± 4.0	10.0 ± 2.5	5.8 ± 1.3
EGFP-VP2-40	0.5 ± 0.1	0.4 ± 0.0	1.8 × 10^-11^	14.0 ± 3.4	9.0 ± 0.7	9.7 ± 0.1
EGFP	0.1 ± 0.0	0.1 ± 0.0	1.2 × 10^-10^	2.0 ± 0.1	2.0 ± 0.1	1.0 ± 0.0

The diffusion times before (τ_D1_) and after (τ_D2_) treatment with 6 M urea at 50°C, diffusion coefficient (*D*1), hydrodynamic radii in the absence (Rh_D1_) and presence (Rh_D2_) of 6 M urea at 50°C, as well as, the number of fluorescent moieties (Nf) present in the EGFP-VP2, EGFP-VP2-14, EGFP-VP2-23 and EGFP-VP2-40 fluorescent proteins and VLPs. The enhanced green fluorescent protein (EGFP) served as a reference.

The number of fluorescent units per capsid was measured for each construct. After incubation of constructs EGFP-VP2, EGFP-VP2-23 and EGFP-VP2-40 in 6 M urea for 15 min at 50°C, the hydrodynamic radii were reduced to approximately 10 nm (Table 1) due to disassembly of the VLPs. A 6 or 10 fold increase in the fluorescent particle numbers was observed, suggesting at least 10, 6 and 10 fluorescent moieties for EGFP-VP2, EGFP-VP2-23 and EGFP-VP2-4 VLPs, respectively (Table 1). Followed by exposure of EGFP-VP2-14 to 6 M urea at 50°C, the fluorescent particle number increased by a factor of 2. These particles showed a diffusion coefficient consistent with a globular protein of about 85 kDa, corresponding to a single EGFP-VP2-14 fusion protein (Table 1 and Fig. 2B).

### Effect of limited proteolysis on the structure of fluorescent proteins

**Figure 7:**
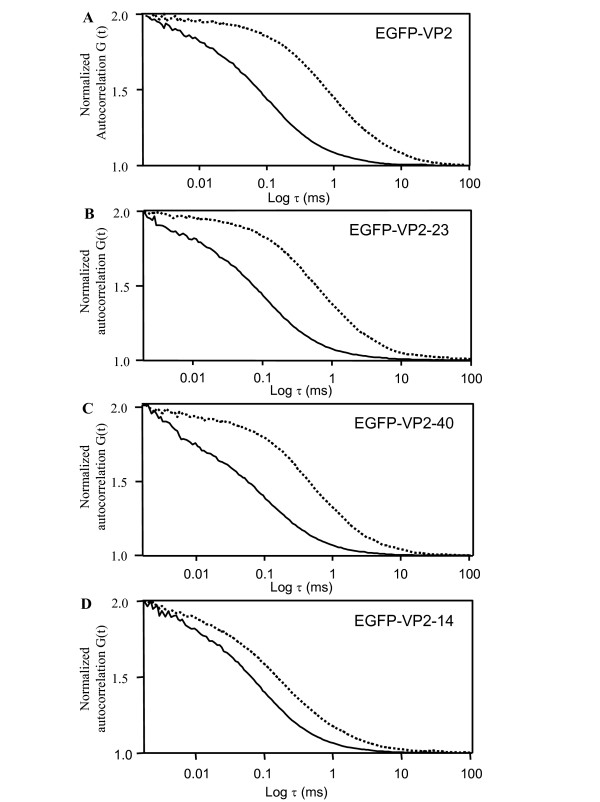
**The effect of trypsin on the fluorescence autocorrelation curves of the fluorescent recombinant proteins**. Normalized autocorrelation curves of EGFP-VP2 **(A)**, EGFP-VP2-23 **(B) **EGFP-VP2-40 **(C) **and EGFP-VP2-14 **(D) **in the absence (...) and presence (—) of 8.3 × 10^-13 ^M trypsin.

The fluorescent fusion constructs, EGFP-VP2, EGFP-VP2-23, EGFP-VP2-40 and EGFP-VP2-14 were characterized by FCS before and after treatment with trypsin. The diffusion times before trypsin treatment (Fig. 7A–C) corresponded well to the size of typical VLPs (Fig. 6, Table 1). It has been previously shown that trypsin can be used for cleaving away the N-terminus of the VP2 protein [24-26]. In the presence of 8.3 × 10^-13 ^M trypsin (5 min), the diffusion times were reduced to the 0.1 ms range. The diffusion times for released particles were the same as compared to the diffusion time of free EGFP, 0.1 ms (Fig. 6). This showed that EGFP was completely released from the surface of the fluorescent VLP for EGFP-VP2, EGFP-VP2-23 and EGFP-VP2-40 (Figs. 7A–C). The diffusion coefficient for EGFP (Table 1) was in agreement with those reported previously [19,27,28]. Further, the diffusion times were clearly lower than the diffusion time of the fluorescent EGFP-VP2-14 protein (0.3 ms; Fig. 6, Table 1), suggesting that the EGFP-VP2-14 proteins are bigger than free EGFP. Together, these results suggest that EGFP was exposed on the surface of the fluorescent VLPs and that EGFP was released by proteolysis in the presence of 8.3 × 10^-13 ^M trypsin (Fig. 7).

## Discussion

Virus-like particles, VLPs, are multimeric structures that are morphologically and structurally very similar to their original viral counterparts. Due to their safety, these types of reagents or particles have been exploited for e.g. antibody detection, as vaccines and antigens and lately also as gene delivery vehicles [23,29-32]. Various manipulations of parvoviral structural proteins in order to understand e.g. structure/function relationships at the molecular/cellular levels have been carried out. These include deletions of [33] and epitope insertions in the loops of the virion [34], N-terminal fusion of foreign antigens to VP1 [35,36], N-terminal insertion and fusion [17,19,37] or deletions of VP2 [33,38], and C-terminal fusions to VP2 [34,37,39].

**Figure 8:**
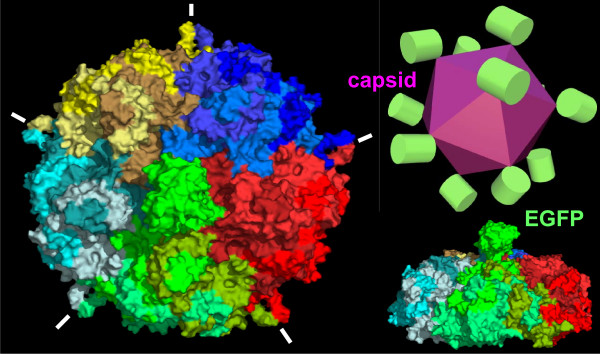
**Models of a fluorescent canine parvovirus virus-like particle**. The model was generated from EGFP-VP2-40, showing 15 subunits of the virus-like particle. EGFP emerges from the 5-fold axis and nestles onto the cannon structure. Molecular model (left) and schematic representation (top right) of the EGFP-VP2-40 recombinant capsid structure. EGFP protrudes through the 5-fold axis cannon structures, indicated by white lines, with the central EGFP domain shown in green. Top and side views of a 15 mer region of the capsid model are shown (left and bottom right, respectively). VP2-40 polypeptides belonging to the same trimers that form single facets of the capsid icosahedron are shown in greens, reds, dark blues, yellows and light blues. The EGFP domain is a continuous polypeptide with one of the three VP2-40 domains in each facet of the icosahedral capsid structure, with the EGFP of the other two VP2-40 polypeptides removed by proteolysis before or during capsid assembly. The EGFP domains could be located at all or some of the twelve cannon structures, depending on proteolysis.

Here, N-terminal deletions of the CPV VP2 protein fused to EGFP i.e. EGFP-VP2-14, EGFP-VP2-23 and EGFP-VP2-40 were produced in insect cells using the baculovirus expression vector system (Figs. 1, 2, 3) in order to study capsid assembly with FCS. The results indicated that all constructs were able to form capsid-like structures (Figs. 3, 4, 5, 6, 7, 8, Table 1) with sizes closely resembling native wild-type CPV [22,33,40], except for the EGFP-VP2-14 construct. This was confirmed by electron microscopy (Fig. 4).

The data obtained from the electron microscopy studies (Fig. 4) and the FCS measurements (Figs. 6, 7) corresponded well with globular VLPs resembling small spherical virions in the range of 25–50 nm in diameter. By comparing all of the constructs, a small, but reproducible deviation in the size and number of the fluorescent fusion proteins present in the fluorescent VLPs was detected by FCS (Table 1). When EGFP-VP2 VLPs were analyzed, the hydrodynamic radius was approximately 17 nm and the approximate amount of fluorescent moieties per particle was 10. The hydrodynamic radius of EGFP alone was 2 nm (Table 1). Deletion of the first 14 amino acids from the N-terminus of VP2 (EGFP-VP2-14) resulted in a drastic change of the hydrodynamic radius from 17 nm to 7 nm with the presence of only 2 fluorescent moieties suggesting that EGFP-VP2-14 lacks the ability of appropriate assembly. This is interesting, since the other two deletions, i.e. EGFP-VP2-23 and EGFP-VP2-40, are similar in size compared to the EGFP-VP2 particles containing the complete VP2 coding sequence (Table 1). The EGFP-VP2-23 construct, however, was slightly bigger than EGFP-VP2, having a hydrodynamic radius of 20 nm with about half (six) the amount of fluorescent particles present in the capsid. However, the hydrodynamic radius of EGFP-VP2-40 was somewhat smaller (14 nm) than that of EGFP-VP2 (17 nm) (Table 1). Further, EGFP-VP2-40 was similar in size to native CPV [2,4,41].

Previous work by Hurtado and co-workers suggested that N-terminal deletions of more than 14 amino acids prevents capsid formation, indicating that residues beyond this point are essential for capsid formation [33]. In addition to their deletions, an insert of two amino acids (L and K; leucine and lysine basic, respectively) prior to the truncated VP2 protein was added. These two amino acid insertions could have had an influence on the structure of the viral protein in order to allow capsid formation. In this study, deletions of 14 amino acids from the N-terminal region of VP2 proteins without any insertions of amino acids, but with a fusion to EGFP appeared not to form VLPs. However, fusions of EGFP to truncated forms of VP2 (-23 and -40) did form VLPs. In addition, VP2-14, VP2-23 and VP2-40 N-terminal deletions could all facilitate capsid assembly. Similarly, it has been demonstrated that most of the N-terminus of human parvovirus B19 VP2, up to 25 amino acids, including the polyglycine region, could be removed without affecting capsid self-assembly, but truncations beyond amino acid 30 were incompatible for either self-assembly or co-assembly with normal VP2 [38]. These data support the claim that the amino acids from the residue 14 (A14) to the residue 23 (S23) of VP2 are important for VLP formation, but that truncations of the CPV VP2 protein could tolerate even deletions of up to 40 amino acids.

Moreover, within our study an average of 10 fluorescent fusion protein units in the EGFP-VP2 and EGFP-VP2-40 VLPs, and 6 in the EGFP-VP2-23 VLPs shows similarities with studies previously reported for the copy number of the VP1 protein within native CPV capsids [33]. Molecular models built in this work and existing structural data support the hypothesis that only one polypeptide may emerge from each five-fold axis of the capsid giving a maximum of 12 EGFP-VP2 fusion protein subunits per capsid (Fig. 8). Due to the fact that the first 37 amino acids of CPV VP2 are not structurally resolved, it is difficult to speculate upon why deletions of the first 14 residues of VP2 results in a structure unable to form VLPs when fused to EGFP. However, it is reasonable to suggest that this area of the peptide (14; A-alanine) is important in CPV VLP assembly. Fusions at the N-terminus of VP2 may play an essential role for such chimeric VLPs being able to display foreign proteins from their icosahedral 5-fold axis. This has been observed before when only two amino acids were attached to a deleted CPV VP2 polypeptide of 14 amino acids [33]. Together this suggests the possibility of replacement of the first 40 amino acids to be used for designing novel vectors for various display purposes e.g. for targeting to specific cell types, without interfering with natural assembly or capsid morphology.

Meyer-Almes and co-workers have quite recently shown that very low enzyme concentrations of target molecules in FCS can be used [42]. The FCS results presented here (Fig. 7) show that single fluorescent VLPs were seen at concentrations of 2 × 10^-9 ^M. In the presence of trypsin at a concentration of only 8.3 × 10^-13 ^M and after a 5 min incubation time, the EGFP moieties were completely released giving one diffusion coefficient identical to that of EGFP. Thus, the results in terms of the amount of fluorescent moieties per fVLP obtained after the enzyme treatment were similar or identical to those obtained after treatment with urea at 50°C (data not shown). In contrast, a previous report on chimeric CPV VLPs has been conducted with equal molar ratio of the protease and the VLPs, 1:1 [24]. Together, the data show that FCS should be attractive in more general terms when molecular assembly mechanisms of different VLPs are under study, e.g. when engineering stable viral vehicles for protein delivery purposes.

## Conclusion

Together, the data presented here show that it was possible to study assembly of a series of truncated and fused CPV VP2 proteins and also to detect deviations in the ability of these proteins to assemble into VLPs using FCS. The non-fluorescent VP2-14, VP2-23, VP2-40 proteins in addition to the full length VP2 -constructs were all able to form VLPs. In addition, the fluorescent proteins of EGFPVP2-23, EGFPVP2-40 and EGFP-VP2 were also able to assemble into VLPs. Interestingly, the VP2-40 construct allowed better adaptation for the fusion polypeptide to be further displayed on the surface of the capsid-like structure. There was some proteolysis, as the average number of fluorescent fusion molecules per particle was 10, the theoretical maximum being 12. The 14 amino acid deletion of VP2 fused to EGFP caused a drastic change in the assembly properties, which was detected by FCS. In conclusion, the results show that FCS provides a novel platform to study assembly of viral proteins and, thus, is a valuable technology that can be utilized for strategic development of VLPs.

## Methods

### Plasmid constructs

The DNA sequences of truncated VP2 genes VP2-14, VP2-23, and VP2-40 were amplified by PCR using pVP2FastBac [19] as a template with 5 sequences starting from the DNA corresponding to 14, 23, and 40 amino acids, respectively, downstream from the N-terminus of VP2. The sense oligonucleotide primers for VP2-14, VP2-23, and VP2-40 were 5'-GGT GGA TCC ATG GTC AGA AAT GAA AGA GC-3', 5'-GCT GGA TCC ATG GGG AAC GGG TC-3' and 5'-GTG GGA TCC ATG TCT ACG GGT ACT TTC AAT AAT C-3', respectively. The anti-sense oligonucleotide primer for VP2-14, VP2-23, and VP2-40 was 5'-CGA GGC GAA TTC TTA ATA TAA TTT TCT AGG TGC-3'. The PCR products of the truncated VP2 genes were digested with *Bam*HI and *Eco*RI and cloned into pFastBacI (Gibco BRL, Grand Island, NY). The resulting plasmids were named pVP2-14FastBac, pVP2-23FastBac, and pVP2-40FastBac. The coding sequence of EGFP was amplified as previously described [19], digested with *Bam*HI and *Bgl*II, and then cloned into the *Bam*HI site of pVP2-14FastBac, pVP2-23FastBac, and pVP2-40FastBac. For EGFP, the sense oligonucleotide primer was 5'-GTC GGA TCC ATG GTG AGC AAG GGC G-3' and the anti-sense oligonucleotide primer 5'-TAA AGA TCT CTT GTA CAG CTC GTC CA-3'. The resulting plasmids were designated pEGFP-VP2-14FastBac, pEGFP-VP2-23FastBac and pEGFP-VP2-40FastBac.

### Recombinant baculoviruses

Two sets of recombinant baculoviruses named *Ac*VP2-14, *Ac*VP2-23, and *Ac*VP2-40 as well as *Ac*EGFP-VP2-14, *Ac*EGFP-VP2-23, and *Ac*EGFP-VP2-40 were generated using the Bac-to-Bac system (Gibco BRL) [43,44]. Generation of the recombinant baculoviruses *Ac*EGFP-VP2 and *Ac*VP2 has been described previously [19,45].

### Production and purification of recombinant proteins

Production and purification of EGFP-VP2, EGFP-VP2-14, EGFP-VP2-23, and EGFP-VP2-40 recombinant proteins was conducted essentially as previously described [19]. In short, 8 × 10^7 ^of S*f*9 cells (Gibco BRL) in a volume of 40 ml HyQ SFX medium (HyClone Inc., Logan, UT) were infected with the recombinant viruses, *Ac*EGFP-VP2, *Ac*EGFP-VP2-14, *Ac*EGFP-VP2-23 and *Ac*EGFP-VP2-40 at a multiplicity of infection (MOI) of 10. At 72 h post infection (p.i.), 500 μl samples were analyzed by SDS-PAGE, immunoblotting and confocal microscopy (see below). Cells were then collected by low speed centrifugation (1 000 × *g*, 10 min, 4°C), resuspended on ice for 15 min in 4 ml of ice cold TENT buffer (50 mM Tris-HCl, 10 mM EDTA, 150 mM NaCl, pH 7.5) containing 0.2% Triton X-100, 2 mM PMSF (phenylmethylsulphonyl fluoride), 10 μg/ml of aprotinin, 10 μg/ml leupeptin and 10 μg/ml pepstatin (Sigma-Aldrich, St. Louis, MI). After clarification (10 000 × g, 20 min, 4°C), 1 ml samples of the supernatants were loaded onto 10 – 40% sucrose gradients in TENT buffer prepared using a Gradient Master™ (BioComp Instruments, Inc. Canada) and 37 ml ultracentrifugation tubes (Beckman Instruments, San Diego, CA). Samples were ultracentrifuged (27 000 × g, 12 h, 4°C) and opalescent or fluorescent bands were detected visually under visual or UV light and extracted. Bands were then diluted in PBS, ultracentrifuged (200 000 × g, 1 h, 4°C), and resultant pellets resuspended in 500 μl ice cold TENT buffer. Alternatively, gradients were fractionated into 1 ml aliquots with a peristaltic pump followed by detection of the recombinant proteins in each fraction by dot blot analysis using anti-CPV capsid antibody (A4E3) or by fluorescence measurements (485 nm excitation, 535 nm emission, 1 s detection time, Wallac VICTOR^2 ^D Fluorometer, Perkin Elmer Life Sciences Inc., MA).

### FCS setup

A confocal fluorescence correlation spectroscope, ConfoCorII (Carl Zeiss, Jena, Germany) was used to carry out the FCS experiments. Focusing was performed in LabTek^® ^II (Nalge Nunc International, Naperville, IL) 8 chamber borosilicate cell culture plates 200 nm above the coated glass surface, and the fluorescence was collected through a Zeiss C-Apochromat 40 × NA 1.2 water immersion objective. A band pass emission filter (530–600 nm) through the same objective was used to filter out emitted photons from the excitation photons.

### FCS analysis of chemically or enzymatically treated recombinant proteins

Preliminary FCS autocorrelation measurements of 10 × 20 s were carried out by diluting the fractions from the sucrose gradient 1:200 to PBS into the FCS sample chamber. A one-component model for the autocorrelation analysis was employed for particle size measurements. The fractions containing particles diffusing with a size corresponding to native VP2 VLPs, having the best fits from the autocorrelation curves, were selected for further analysis. For EGFP-VP2-14, no sucrose gradient fractions contained VLPs detectable by immunoblotting (Fig. 5) or contained particles with the size of VLPs when detected by FCS. Instead, the fractions having the highest count rate in FCS, 30–35, were pooled for further analysis. For particle number measurements, fractions 17–20 containing fluorescent VLPs were chosen and further incubated for 15 min at 50°C in the presence of 6 M Urea followed by FCS analysis. In addition, the corresponding samples (capsids) were diluted to 2 nM concentrations followed by treatment with 8.3 × 10^-13 ^M trypsin (5 min) and then analyzed by FCS. Averages and the standard deviations for the diffusion times were measured and calculated. Diffusion times were used to calculate the diffusion coefficient *D *for the sample, using the ratio of the diffusion time of Rhodamine 6G (Rh6G) dye having a diffusion coefficient 2.8 × 10^-6 ^cm^2 ^s^-1^, and the diffusion time of the sample [46]. FCS analysis was carried out in 15 × 40 s measurements repeated 6 times for each sample. All samples were stored on ice before measurements, and equilibrated 10 min at room temperature (20°C) prior to FCS analysis. Hydrodynamic properties of particles were calculated by using the Stokes-Einstein equation *D *= kT/6πrη, where *D *is the diffusion coefficient, *r *is the hydrodynamic radius, η is the viscosity of the solvent, T is the temperature and k is Boltzman's constant.

### SDS-PAGE and immunoblotting

Samples [47] were separated on 12% slab gels and transferred to nitrocellulose sheets (Schleicher & Schuell BioScience, Inc., Keene, NH) for immunoblot analysis. Proteins were identified with rabbit polyclonal anti-VP2 antibodies (Cornell #2 antibody, kind gift from Dr. Colin Parrish), a mouse monoclonal anti-CPV capsid antibody A4E3 (dot-blot), or with rabbit polyclonal anti-GFP antibodies (Promega, Madison, WI). Proteins were visualized with AP-conjugated goat anti-rabbit IgG (Promega) and with NBT (nitro blue tetrazolium) and BCIP (5-bromo-4-chloro-3-indolylphosphate; Sigma-Aldrich). Molecular weight markers were from BioRad.

### Immunolabeling of S*f*9 cells

Infected insect cells were collected by low speed centrifugation (800 × g, 1 min, RT), washed twice with PBS (pH 7.4) prior to fixation in 50 μl of 4% PFA-PBS (20 min, RT) and then pelleted (10 000 × g, 1 min, RT). Before immunostaining, cells were rinsed twice with 50 μl of 0.15% glycine in PBS with a centrifugation step (10 000 × g, 1 min, RT) between each wash. Fixed cells were permeabilized (1% BSA, 0.1% Triton X-100 and 0.01% sodium azide in PBS) for 20 min at RT and pelleted (10 000 × g, 1 min, RT). A mouse monoclonal anti-capsid antibody, A4E3 (kind gift from Dr. Colin Parrish) was used to identify VP2. After incubation with the anti-capsid antibody, cells were washed 3 times in 50 μl PBS and the viral proteins probed with a fluorescently conjugated secondary antibody (goat AlexaFluor^® ^633 anti-mouse antibody, Molecular Probes, Eugene, OR). After fluorescent staining, cells were washed twice with 50 μl PBS and pelleted (10 000 × g, 1 min, RT). Finally, cells were embedded in 2–7 μl of MOWIOL-DABCO (30 mg/ml; Sigma-Aldrich) and cover slips were left for 2–24 h at 4°C before examination under a confocal laser scanning fluorescence microscope (Axiovert 100 M, LSM510, Carl Zeiss, Jena, Germany) with excitation and emission settings appropriate for the dyes used according to the manufacturer's instructions.

### Electron microscopy

Specimens for electron microscopy (EGFP-VP2, EGFP-VP2-14, EGFP-VP2-23, EGFP-VP2-40, VP2, VP2-14, VP2-23, and VP2-40) were prepared from the samples obtained by sucrose gradient purification. Resuspended samples (6 μl) were applied to carbon coated copper grids and left to stand for 1–2 min at RT. After excess liquid was blotted away, grids were negatively stained with 6 μl 2% potassium phosphotungstate, pH 6. Excess liquid was further removed and grids left to dry at RT. Samples were examined at 60 kV under a JEOL JEM-1200 EX transmission electron microscope (Jeol Ltd. Tokyo, Japan).

### Molecular modelling

A molecular model of EGFP-VP2-40 was constructed based on the crystal structures of EGFP (PDB 1EMF) and CPV VP2 (PDB 4DPV) with a surrounding 14 subunits of VP2 (PDB 1C8D), to form a 15-mer around one 5-fold axis, through which the N-terminus of VP2-40 protrudes, carrying the EGFP domain. Models were built in Insight II (Accelrys) and graphics were rendered using Pymol 0.98 (Delano, W.2. The Pymol molecular Graphics System 2002).

## Authors' contributions

LG was responsible for drafting and finalizing the manuscript. LG planned and performed the genetic baculovirus constructs and generation of the recombinant baculoviruses. LG was also responsible for the production, purification and analysis of the recombinant proteins, as well as, electron and confocal microscopy data. JT helped with purification and analysis of the recombinant proteins, carried out all the FCS experiments and helped with drafting the manuscript. OV helped in performing the electron microscopy experiments, whereas TS and CC assisted in cloning and generation of the recombinant baculoviruses. AM contributed in construction of the second set of baculoviruses and EK helped with production and purification of recombinant proteins. DW designed the structural models of the recombinant capsid structures and assisted in the interpretation of results. MV participated in the FCS data collection, contributed to the interpretation of the results and finalizing the manuscript. CO-B provided guidance regarding the experimental design as well as in the preparation and finalizing of this manuscript.
